# Assessing the role of synchronization and phase coherence in neural communication comparing cortical recordings and integrate-and-fire network models

**DOI:** 10.1186/1471-2202-14-S1-P306

**Published:** 2013-07-08

**Authors:** Daniel Chicharro, Christoph Kayser, Stefano Panzeri

**Affiliations:** 1 Center for Neuroscience and Cognitive Systems, Istituto Italiano di Tecnologia, Rovereto (Tn), 38068, Italy; 2 Institute of Neuroscience and Psychology, University of Glasgow, Glasgow, G12 8QB, UK; 3 Bernstein Center for Computational Neuroscience, Tübingen, 72076, Germany; 4 Max Planck Institute for Biological Cybernetics, Tübingen, 72076, Germany

## 

Cognitive functions likely require that the routes of neural communication can be flexibly modulated. A proposed mechanism for modulating the effective strength of the connections in the neural dynamics relies on band specific neural synchronization and phase relations [1]. Evidence of the modulation of neuronal interactions through the phase relation of rhythmic activity in the gamma band was provided in [2]. Further evidence based on the analysis of a network of integrate-and fire neurons [3] has shown that the phase relation also modulates information transfer and is not specific of the gamma band. Here we combine the study of experimental recordings of local field potentials (LFP's) and multiple-unit activity (MUA) together with model data to better understand the origins of the phase-dependent modulation of interactions. Recordings include spontaneous activity and natural stimulus-driven activity in the monkey visual cortex V1 [4], as well as natural-stimulus driven activity in monkey auditory cortex [5]. Simulations were obtained extending the recurrent network of integrate-and-fire neurons used in [6] to model the connectivity between two different brain areas.

We address some open questions regarding the generation, generality, and mechanistic nature of the phase-dependent modulation. We obtained, for any frequency band, the instantaneous power in each area (reflecting the local neural synchronization), and the instantaneous phases. We analyzed how the power correlation is modulated by the phase relation with a 1ms resolution, in contrast to the hundreds of milliseconds in [2]. We found that this modulation is accompanied by changes in the magnitude of the power of each area separately. Accordingly, we evaluated the role of the power determining the degree of phase coherence and thus the existence of a preferred phase relation. We found that the optimal phase relation associated with maximal power correlation always corresponds to the preferred phase relation for large powers. These results are not frequency band specific and were reproduced with model data, using both unidirectional and bidirectional connections, as well as both excitatory-excitatory and excitatory-inhibitory connections. Our analysis suggests that the degree of local neural synchronization that determines the power of a given rhythm in each of the interacting areas plays a role to be considered together with the one of the phase relations as part of the mechanisms that modulate dynamically the effective strength of the connections.
